# Genome Sequence of the *Siphoviridae* Staphylococcus aureus Phage vB_SauS_BaqSau1

**DOI:** 10.1128/MRA.00147-20

**Published:** 2020-04-09

**Authors:** Dayan Lozano-Solano, Jhonnatan Reales-González, Heath W. Catoe, Raul R. Raya, Antonio J. Acosta-Hoyos

**Affiliations:** a Universidad Simón Bolívar, Facultad de Ciencias Básicas y Biomédicas, Unidad de Genética y Biología Molecular, Barranquilla, Colombia; b Universidad del Atlántico, Departamente de Biología, Barranquilla, Colombia; c University of Miami Miller School of Medicine, Miami, Florida, USA; d Centro de Referencia para Lactobacilos (CERELA), San Miguel de Tucumán, Argentina; Queens College

## Abstract

Here, we report the genome sequence of a *Siphoviridae* phage named vB_SauS_BaqSau1 (BaqSau1), infecting Staphylococcus aureus. Phage BaqSau1 was isolated from a sewage water treatment plant in Sahagún, Córdoba, Colombia. It has a double-stranded DNA (dsDNA) genome of 44,384 bp with 67 predicted genes, including a lysin containing a CHAP (cysteine, histidine-dependent amidohydrolase/peptidase) domain.

## ANNOUNCEMENT

Staphylococcus aureus is a common pathogen that causes a wide range of infections in humans and animals. The Centers for Disease Control and Prevention (CDC) established the dissemination of methicillin-resistant Staphylococcus aureus (MRSA) as a worldwide health problem due to its resistance to multiple antibiotics (www.cdc.gov/drugresistance/index.html), and the World Health Organization (WHO) recently included MRSA in a high-priority list of bacteria for which new antimicrobial strategies are urgently needed (https://www.who.int/medicines/publications/WHO-PPL-Short_Summary_25Feb-ET_NM_WHO.pdf). Several characteristics of phages, for example, specificity, selectivity, self-limiting replication, and constant evolution, make them a promising alternative to antibiotics for treatment of bacterial infections (1). Here, we describe the lysogenic Staphylococcus aureus phage vB_SauS_BaqSau1 (BaqSau1).

**FIG 1:**
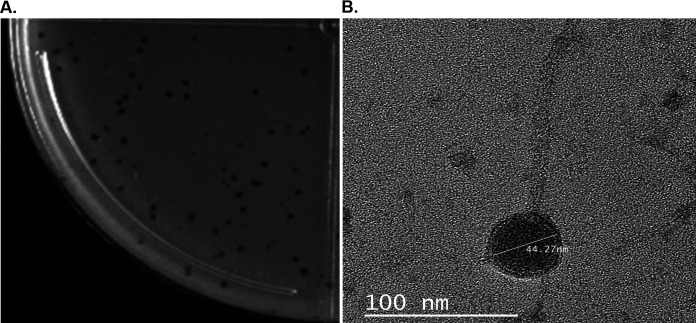
Plaque appearance (A) and virion morphology (B) of bacteriophage vB_SauS_BaqSau1. The plaques were obtained as described in the text. For electron microscopy, a high-titer lysate was applied on Formvar-coated grids, negatively stained with 2% uranyl acetate, and imaged with a Zeiss EM-109 transmission electron microscope (Carl Zeiss AG); the capsid size is approximately 44 nm.

S. aureus strain RN4220 (2) was used to isolate bacteriophage BaqSau1 from wastewater collected in Sahagún, Córdoba, Colombia, using a 24-hour enrichment process (3), and then to propagate it on tryptic soy (TS) broth or TS agar supplemented with 0.05 mM MgSO_4_ using the double-layer overlay technique (4) (Fig. 1A). Genomic DNA was extracted using the PureLink viral RNA/DNA minikit (ThermoFisher Scientific) according to the protocol described by the Center for Phage Technology at Texas A&M University (https://cpt.tamu.edu/phage-links/phage-protocols/). Whole-genome sequencing was done by ACGT, Inc. (USA). The DNA was fragmented by ultrasonication to an average target fragment size of 550 bp and used for constructing a sequencing library using the NEXTflex rapid DNA sequencing kit. The final library was sequenced with the Illumina MiSeq v3 flow cell instrument, which generated 6,381,090 300-bp paired-end raw reads. The adapter and low-quality sequences (Q < 30) were trimmed, and short reads (<50 bp) were filtered out using Trimmomatic software v0.36 using default configurations (5). The trimmed reads were *de novo* assembled using SPAdes v3.11.1 (6), resulting in a contig with 86.4× fold coverage. The final contig was annotated with RAST (7) using the *PhiETA2* genome as the reference (taxonomy identification number 326036). The coding DNA sequences (CDSs) and putative functions were predicted by RAST and further analyzed using HHpred (8). Average nucleotide identity (ANI) analysis was done with CLC Genomics Workbench v20.0 (Qiagen) using default configurations.

BaqSau1 is a new member of the *Siphoviridae* family (Fig. 1B); its genome consists of a double-stranded DNA molecule of 44,384 bp with an overall G+C content of 34.05%. Average nucleotide identity (ANI) (9, 10) analysis of BaqSau1 with other *Staphylococcus* phages revealed that it has over 90% nucleotide sequence identity with members of the *Phietavirus* genus (11). Genome analysis revealed a total of 67 coding DNA sequences (CDSs), of which 34 had a predicted function. The functional CDSs were categorized into 4 clusters of functionally related putative genes as follows: (i) phage structure and packaging proteins, 21 CDSs; (ii) DNA replication and regulation proteins, 6 CDSs; (iii) life cycle proteins, 5 CDSs; and (iv) lysis proteins, 2 CDSs. Potential key virulence factors of S. aureus were found in 3 CDSs, as well as a CDS encoding a superantigen pathogenicity island SaPI, which provides resistance to beta-lactamases and the Panton-Valentine leukocidin (PVL) toxin gene (12, 13). Open reading frame (ORF) 29 codes for an integrase with a C-terminal integrative and conjugative element from *Bacillus subtilis* (ICEBs)-like catalytic domain (cd01189). The lytic enzymes identified were a class II holin (ORF 26) and an *N*-acetylmuramoyl-l-alanine amidase endolysin (ORF 27) containing a CHAP (cysteine, histidine-dependent amidohydrolase/peptidase) domain (14) similar to LysK from staphylococcal bacteriophage K, a lytic phage against MRSA (15).

Finally, we conclude that BaqSau1 is a new member of the temperate *Siphoviridae* belonging to the *Phietavirus* genus, containing an endolysin with possible antibacterial activity.

### Data availability.

The genome sequence of phage BaqSau1 was deposited in GenBank under the accession number MK658834. The raw sequence reads have been submitted to the NCBI SRA under accession number PRJNA610274.
