# Correction for Lozano-Solano et al., “Genome Sequence of the *Siphoviridae* Staphylococcus aureus Phage vB_SauS_BaqSau1”

**DOI:** 10.1128/MRA.01101-20

**Published:** 2020-12-03

**Authors:** Dayan Lozano-Solano, Jhonnatan Reales-González, Heath W. Catoe, Raul R. Raya, Antonio J. Acosta-Hoyos

**Affiliations:** a Universidad Simón Bolívar, Facultad de Ciencias Básicas y Biomédicas, Unidad de Genética y Biología Molecular, Barranquilla, Colombia; b Universidad del Atlántico, Departamente de Biología, Barranquilla, Colombia; c University of Miami Miller School of Medicine, Miami, Florida, USA; d Centro de Referencia para Lactobacilos (CERELA), San Miguel de Tucumán, Argentina; e Universidad del Valle, Cali, Colombia

## AUTHOR CORRECTION

Volume 9, no. 15, e00147-20, 2020, https://doi.org/10.1128/MRA.00147-20. Page 1: The author affiliations should appear as shown in this correction.

